# Is Hypothyroidism and Hypogonadism an Issue After Aneurysmal Subarachnoid Hemorrhage-An Institutional Experience?

**DOI:** 10.5812/ijem.8241

**Published:** 2013-07-01

**Authors:** Nayil Khursheed, Altaf Ramzan, Yawar Shoaib, Iftikhar Bashir, Abrar Wani, Alam Shafiq

**Affiliations:** 1 Department of Neurosurgery, Sheri-Kashmir Institute of Medical Sciences, Kashmir, India; 2 Department of Endocrinology, Sheri-Kashmir Institute of Medical Sciences, Kashmir, India

**Keywords:** Hypothalamic Diseases, Hypogonadism, Hypothyroidism

## Abstract

**Background:**

The incidence of hypopituitarism in aneurysmal subarachnoid hemorrhage ranges from 0% to 45%. Also the screening for hypopituitarism in survivors of aneurysmal SAH is not a routine. This has led to a controversy in the management of such patients.

**Objectives:**

The aim of the study was to evaluate the endocrine profile of our patients who had presented with aneurysmal SAH.

**Patients and Methods:**

This was a prospective study conducted over a period of three years in patients of aneurysmal subarachnoid hemorrhage. The serum samples for levels of free T4, free T3, TSH, prolactin, FSH, LH and testosterone were analyzed at the time of admission and at a follow-up period between 9-12 months. Patients with known endocrine abnormalities, liver or kidney disease and patients with hemodynamic abnormalities were excluded from the study. Abnormalities in levels were noted and a comparative analysis of the hormone levels between the 2-time periods was done. A total of 73 patients were enrolled in the study.

**Results:**

Serum prolactin was raised in 17.80% (13/73) and FSH, LH and testosterone levels were reduced in 12.32% (9/73) of patients in the acute phase at admission. After 9 months follow-up, serum prolactin normalized in all except one patient and in all the males, testosterone level increased significantly. Two patients (3%) developed central hypothyroidism on follow-up.

**Conclusions:**

Chronic hypothyroidism and hypogonadism is not an issue in aneurysmal SAH patients

## 1. Background

The incidence of hypopituitarism in aneurismal subarachnoid hemorrhage (SAH) has been reported from 0% to 45% (1-5). Presently screening for pituitary dysfunction in aneurismal SAH is not a routine practice in neurosurgical centers. However; this variation has raised many questions ranging from whether undiagnosed pituitary dysfunction contributes to the long term morbidity in survivors of SAH (6) or whether the screening for patients of aneurismal SAH is a futile exercise (1).

## 2. Objective

The study was undertaken with a resolve to elucidate the incidence and severity of acute and chronic pituitary dysfunction in patients suffering from aneurismal SAH.

## 3. Patients and Methods

This prospective study was conducted in the Departments of Neurosurgery and Endocrinology, Sher-i-Kashmir Institute of Medical Sciences, Kashmir, India. The study period was from January 2009 to December 2011. All the patients who had CT scan or lumbar puncture proved subarachnoid hemorrhage and the aneurismal source of SAH was later confirmed by CT angiography were screened for inclusion and exclusion criteria. Patients who were on hormone supplements, previous subarachnoid hemorrhage, chronic renal failure, chronic liver disease like cirrhosis and patients who were hemodynamically unstable were excluded from the study. Out of a total of 108 patients, 73 were finally enrolled in the study. A written consent was signed by all patients or the next-of-the kin. The fasting serum levels of free T4, free T3, TSH, prolactin, FSH, LH and testosterone levels were done on two occasions for all the patients. Testosterone levels were measured only in male patients. Samples were sent at the time of admission and then on follow-up between nine months to one year in neurosurgical outpatient clinic. Growth hormone and cortisol levels were not sent as the facilities for their stimulation tests did not exist in our laboratory. Neurological grade of the patients was assessed by World Federation of Neurological Surgeons (WFNS) Scale (7). CT grading of SAH was done by Fisher’s grading (8). All patients were treated surgically. There were 84 aneurysms in 73 patients. Clipping and wrapping of the aneurysms were done in 82 and 2 patients, respectively. Surgery was performed between two days to nine days of ictus. Early surgery (within 72 hours of ictus) was performed in 28 patients. Mean duration of surgery was 248 min (range 83-412 min). Temporary clip was applied for 51 aneurysms on the parent artery. In patients of anterior communicating (Acom) artery aneurysm with hydrocephalus, the lamina terminalis was opened at surgery. Two patients with middle cerebral artery (MCA) aneurysms developed communicating hydrocephalus at follow-up for which ventriculo-peritoneal shunts were put. All patients were managed in intensive care unit from the time of admission for at least 72 hours after surgery. Management in the intensive care unit included invasive monitoring of blood pressure and central venous pressure. Facilities for measurement of pulmonary wedge pressure were not available. During the hospital stay steroids were not used.

### 3.1. Definition of Abnormalities

Central hypothyroidism was defined as serum free–T4 < 4ug/dL associated with an inappropriately low TSH. Hyperprolactinemia was defined as basal level greater than the normal reference range as shown the Table 2. Hypogonadotropic hypogonadism was defined in men as low serum testosterone associated with low LH, in post-menopausal females as inappropriately low gonadotropins and in pre-menopausal females as low estradiol associated with inappropriately low gonadotropins. However, in our study, there were no pre-menopausal females hence estradiol levels were not done. ADH assays were not done as none of our patients reported polyuria or polydipsia.

### 3.2. Statistical Analysis

Statistical analysis was performed by using the statistical package for social sciences (SPSS, version 19, Illinois, Chicago). Categorical and continuous variables were measured by Fishers exact test and paired t-test respectively. P value of 0.05 and below was taken as statistically significant.

## 4. Results

There were 37 males and 36 females; the mean age was 56 + 13.5 years in our study. The general patient characteristics are given in Table 1.

### 4.1. Hormonal Assays at Admission

Serum prolactin was raised in 17.80% (13/73) and FSH, LH and testosterone levels were reduced in 12.32% (9/73) of patients in the acute phase at admission. Free T4, free T3 and TSH levels were normal in all the patients at admission.

### 4.2. Hormonal Assays at Follow-up

The prolactin levels had normalized in all the patients except in one patient of Acom-artery aneurysm in whom there still was mild elevation of prolactin (36.2 ng/mL). Free T4 and TSH levels were reduced in two patients of Acom-artery aneurysm. FSH, LH and testosterone levels had also normalized in all the patients. When the mean hormonal levels in the acute phase and at follow- up were compared (Table 2), there was a notable statistically significant increase in testosterone levels in males and a significant decrease in prolactin levels in all patients (P value = 0.03, df = 1, CI = 0.018-0.711) and (P value = 0.01,df = 1,CI = 0.046-1.270) respectively. There was no correlation between hormone levels and parameters viz. gender, hypertension, smoking, Fisher grade and hydrocephalus (P > 0.05). However, we noticed that patients whose neurologic grade was poor at admission, had higher incidence of hyperprolactinemia at the same time (P = 0.03). New onset central hypothyroidism was observed in two patients at follow-up. Except for this, no other hormone deficiency evolved in this period. There were no premenopausal women in our study.

## 5. Discussion

The prevalence of pituitary dysfunction after aneurismal SAH has been reported up to 45% (5, 6) of cases. Jenkis et al. (9) was the first to report on pituitary dysfunction in aneurismal SAH. Later, Kelly et al. (10) in a series of his patients which included head injury and aneurismal SAH also reported pituitary dysfunction. Tanriverdi F et al. (6) found hyperprolactinemia in 22.7% of patients with aneurismal SAH in their acute-phase of illness. Prolactin levels normalized in all subjects of the same survey in one year period of follow-up. We also had a similar experience: prolactin levels had increased in 13 of our patients (17.80%) in the acute phase of illness and only one patient on follow-up period showed mild elevation of prolactin levels. We also noticed that the mean hormonal levels of prolactin were raised at admission as compared to the levels at follow-up and that the levels of testosterone in the acute stage were lower and there was a significant increase in testosterone levels at follow-up. Almost similar observations were made by Tanriverdi F et al. (6) who noted statistically significant changes in the levels of prolactin, testosterone and TSH in the follow-up period as compared to the hormone levels in the acute phase. Among hormones, a high incidence of dysfunction has been seen with the growth hormone and cortisol (4, 11-13). The exact mechanism of it is not yet certain and theories put forward are structural hypothalamic pituitary injury, adaptive mechanism to acute illness, raised intracranial pressure andedema around the hypothalamic -pituitary axis (1). Kreitschmann-Andermahr I et al. (2) in a study of 40 cases of aneurismal SAH found correlation between fenestration of lamina terminalis at surgery and endocrine disturbances at follow-up. They speculated that opening of the lamina terminalis may disrupt neuroendocrine pathways. However, the number of the patients in whom lamina terminalis was not opened during surgery was too low to decide whether this really is a factor. We in our series did not notice any correlation between fenestration of lamina terminalis and hormonal disturbance at follow-up. It has been postulated that physiological response to acute stress causes hyperprolactinemia, central hypothyroidism and hypogonadism. This could be explained by the fact that acute illness actually results in variable degrees of changes in the metabolism of hormone-binding proteins which may vary from patient to patient and hence may reflect upon the levels of hormones. Studies which have reported these changes have been carried out mostly in polytrauma, septic shock and renal failure (1-3, 10, 11, 14-20). It is possible that the response of neurohypophysis to such conditions may be different than the response to aneurismal SAH and that, possibly explains why our patients behaved differently. In a study by Agha A et al. (11) on 50 patients of head injury, hyperprolactinemia was found in 52% (26/50) patients and hypothyroidism in 4% (2/50) patients. Aimaretti G et al. (14) and Dimopoulou I et al. (12) noticed chronic gonadotropin deficiency in 12.5% and 6.25% of patients respectively. However in contrast Tanriverdi F et al.(6) and Kreitschmann-Andermahr I et al. (2) did not observe gonadotropin deficiency at 12 month period after SAH. These conflicting reports of long term pituitary hormone deficiencies in patients of aneurismal SAH are also possible because of the varying criterias and stimulation tests used for the diagnosis and it also depends upon the time interval that has elapsed from the primary event of aneurismal bleeding and the evaluation of hormonal assay (1). As for example, Aimaretti G et al. (14) while evaluating patients of SAH at a three month follow-up period, found gonadotropin deficiency in 12.5% and TSH deficiency in 7.5%. In our study we evaluated patients at nine months to one year period after the SAH and found no chronic gonadotropin deficiency and TSH deficiency was seen in only 2.73% of our patients. This reflects the fact that pituitary gland recovers with time. Another reason that in our study hormonal disturbances did not have a correlation with the clinical grade could be attributed to the fact that most of the patients in our study were in good neurologic grade and we had only very few patients in poor neurologic grade who came to our outpatient clinic at one year follow-up period. This patient selection bias must have reduced the power of the statistical analysis. Klose M et al. (1) in a study of 62 patients of aneurismal SAH also reported no incidence of pituitary hormone dysfunction including growth hormone and cortisol and has disregarded the myth of chronic hypopituitarism in aneurismal SAH. The severity of the acute illness also correlates with the prolactin levels as various studies have shown that prolactin levels increase more in patients who have poor neurological score at admission (4, 21).

### 5.1. Limitations of the Study 

The present study has not evaluated the more cortisol and growth hormone levels which are supposed to get involved more than the other hormones; however it has reasonably endeavored to elucidate the temporal fate of other pituitary hormones in aneurismal subarachnoid hemorrhage. Even though pituitary dysfunction is common in acute illnesses including aneurismal SAH, hypothyroidism and hypogonadism is not an issue in aneurismal SAH patients, however central hypothyroidism may develop in some patients.

**Table 1.** Clinicoradiologic Profile of Patients (n = 73)

Clinicoradiologic Profile	
	No.
**Gender**	
Females	36
Males	37
**Hypertension**	39
**Smokers**	19
**WFNS grade**	
1	9
2	27
3	16
4	13
5	8
**Fisher grade**	
1	4
2	12
3	43
4	14
**Aneurysm location**	
A-com artery	43
MCA	29
DACA	5
ICA bifurcation	4
P-com artery	3
Intra-op rupture	12
Fenestration of lamina terminalis	19
**Hydrocephalus**	21

**Table 2.** Mean Hormone Levels at Two Different Stages

Hormones	Normal Range in Our Lab	At Admission, Mean	At Follow-up, Mean
**Prolactin, ng/mL**	1-27 (females)	17.91 ± 1.87	7.2 ± 0.75 ^a^
	1-20 (males)		
**TSH, uIU/mL**	0.5-6.5	3.39 ± 0.35	4.92 ± 0.87
**fT3, ng/mL**	0.70-2.50	2.47 ± 0.24	2.96 ± 0.23
**fT4, ug/dL**	4.0-13.0	11.25 ± 1.22	12.65 ± 0.57
**FSH (female, IU/L)**	27.0-129.0 (post-menopausal)	54.51 ± 9.26	56.93 ± 9.97
**LH (female, IU/L)**	29.0-120.0 (post-menopausal)	42.64 ± 8.50	45.67 ± 8.57
**FSH (male, IU/L)**	1.0-10.0	4.90 ± 0.91	5.75 ± 1.02
**LH (male, IU/L)**	1.0-10.0	4.36 ± 0.74	4.97 ± 1.10
**Total testosterone (male, ng/dL)**	250-1500 (up to 49 years)	196.75 ± 31.50	250.64 ± 36.82 ^a^
	180-700 (beyond 49 years)		

^a^ Statistically significant
